# Metabolites of Microbial Origin with an Impact on Health: Ochratoxin A and Biogenic Amines

**DOI:** 10.3389/fmicb.2016.00482

**Published:** 2016-04-08

**Authors:** Pasquale Russo, Vittorio Capozzi, Giuseppe Spano, Maria R. Corbo, Milena Sinigaglia, Antonio Bevilacqua

**Affiliations:** Department of the Science of Agriculture, Food and Environment, University of FoggiaFoggia, Italy

**Keywords:** safety, biogenic amines, Ochratoxin A, prevention, correction

## Abstract

Safety and quality are significant challenges for food; namely, safety represents a big threat all over the world and is one of the most important goal to be achieved in both Western Society and Developing Countries. Wine safety mainly relies upon some metabolites and many of them are of microbial origin. The main goal of this review is a focus on two kinds of compounds (biogenic amines and mycotoxins, mainly Ochratoxin A) for their deleterious effects on health. For each class of compounds, we will focus on two different traits: (a) synthesis of the compounds in wine, with a brief description of the most important microorganisms and factors leading this phenomenon; (b) prevention and/or correction strategies and new trends. In addition, there is a focus on a recent predictive tool able to predict toxin contamination of grape, in order to perform some prevention approaches and achieve safe wine.

## Introduction

Safety is a challenge for consumers; wine safety relies upon a complex equilibrium from good manufacturing practices, quality of raw materials, fermentation, and post-fermentation events. An outbreak in wine safety generally results in the recovery of a wide variety of harmful compounds with a strong biological activity on human health (carbamate, amines, mycotoxins, heavy metals and residues from wrong production practices, methanol etc.). This paper offers an overview on wine safety with a special focus on some metabolites of microbial origin, namely Ochratoxin A (OTA) and biogenic amines (BA), as the number of papers dealing with these compounds has significantly increased in the last decade, due to an increased awareness on the effects on health and well-being.

Moreover, the choice of focusing on OTA and BA also relies on some other factors, i.e., (i) the origin (OTA from fungi and BA generally from bacteria); (ii) the time of production (pre-harvest and/or pre-fermentation for OTA and throughout fermentation or in the post-fermentation phase for BA); (iii) the molecular weight (lower for BA and higher for OTA); (iv) the increasing prevalence in human outbreaks (Spano et al., 2010; Petruzzi et al., 2014e).

Both BA and OTA are used as topics to address safety issues raised by microorganisms and show how uncontrolled microbial dynamics both on grape and must/wine could lead to significant threats. BA and OTA are described in relation to some traits, like origin, effects on health and correction/prevention strategies, in order to offer a deep insight on the literature and pinpoint the most recent advances on these compounds.

## Pre-Harvest Challenge: Grape and Wine Contamination by Ochratoxin A

Ochratoxin A is produced by *Aspergillus* spp. and *Penicillium* spp. and derives from 3,4-dihydrocumarin linked to an amide bond with an amino group of L-β-phenylalanine (Peraica et al., 1999). It can be recovered in a variety of foods, including cereals, grapes, cocoa, coffee, and spices; its presence in alcoholic beverages is mainly in red wine followed by rosé and white wines (Battilani et al., 2006; Bellver Soto et al., 2014). Although it is found throughout the world, European and African regions are the most affected by this compound (Bellver Soto et al., 2014). Several authors conducted a survey to asses OTA contamination in European wines (Italy, Spain, Greece, Hungary, Croatia) and found it in 60–80% samples, although the median value was strongly variable (from 0.007 to 2.79 ng/ml; Bellver Soto et al., 2014).

Ochratoxin A is a great threat for humans, because it accumulates in several tissues in the body and is classified by the International Agency for Research on Cancer [IARC] (1993) in the group 2B (possible human carcinogen). Kidney is its main target, and hereby causes Balkan endemic nephropathy (BEN), chronic interstitial nephritis, and karyomegalic interstitial nephritis (Simon, 1996). In the kidney, OTA mainly impairs proximal tubular functions and causes glucosuria, enzymuria, and a decrease in the transport of *p*-aminohippuric acid (PAH), a prototypical renal organic anion (Gekle and Silbernagl, 1993, 1994; Dahlmann et al., 1998). The presence of OTA in blood from healthy humans confirms a continuous and widespread exposure (Thuvander et al., 2001; Sangare-Tigori et al., 2006), thus the Scientific Panel on Contaminants in the Food Chain from the European Food Safety Authority [EFSA] (2006) set OTA Tolerable Weekly Intake (TWI) to 120 ng/kg body weight.

A new tool for risk assessment of OTA is predictive mycology, focusing on the development of some model to predict fungal growth and inactivation, such as the theory of the Design of Experiments (Dagnas et al., 2014; Ioannidis et al., 2015; Burgain and Dantigny, 2016), equations to predict the germination of fungal spores (Kalai et al., 2014) or the production of mycotoxins (Aldars-García et al., 2015).

Recently, Battilani and Camardo Leggieri (2015) developed and designed a conceptual model for the dynamic simulation of OTA production on grape berries by *Aspergillus carbonarius*; they used some primary variables (overwinter inoculum, spores on berries, germinated spores, growth on berries, infected berries, colonized berries, and OTA index), intermediate variables (berry status; growth stage of berries), parameters (air temperature, relative humidity, rainfall, *a*_w_, pest and disease) and rates (dispersal, germination, growth, infection, colonization, and OTA production). The variables represent the status of the fungus at any time and OTA-index represents the most important output (OTA contamination); the flow from a status to another is driven by known parameters (temperature, relative humidity etc...) or by intermediate variables derived from crop or weather data. The rates were described by Battilani and Camardo Leggieri (2015) as a kind of valve and are represented by a mathematical function ranging from 0 to 1, with 1 intended as the rate occurring at the optimal conditions and the flow to the next status stopped when the rate is 0. The most important output of this function is a risk model able to build a cumulative function, showing OTA accumulation on berries at any stage and predict if grape is under or over the legal limit and a correction strategy is required. The model was based on the data of a survey conducted in some Italian regions (Apulia, Emilia Romagna) and was preliminary validated with some confirmatory surveys. To our knowledge, this is the only attempt of predictive mycology applied to OTA; therefore, a possible way for innovation could be the design of simple software/Excel file able to predict the risk associated with OTA in grape, must and wines, in order to avoid economic losses due to the toxin.

### Prevention and Correction Strategies

The presence of OTA in the grape can be shifted from grain grapes to wine during fermentation. OTA levels depend on different factors such as vineyard location (latitude), weather (rain, temperature, and relative humidity in the vineyards), period of harvest, pesticide treatments, and wine fermentation, with a strong impact of the duration of grape maceration. The European Union allows a maximum limit for OTA in wine of 2 ng/g (Bellver Soto et al., 2014).

Quintela et al. (2013) extensively reviewed physical, chemical and biological approaches to perform the decontamination of OTA. The dilution of contaminated must is strictly forbidden by EU (Commission Regulation 1881/2006; European Commission [EC], 2006) and few approaches are feasible at industrial levels, like the removal of mouldy grapes or bunches (Rosseau, 2004), the repassage of contaminated must or wines over grape pomaces having no or little OTA contamination (Solfrizzo et al., 2010), pressing the pomace at 80 atm, filtration, heat treatment on a hot plate at 55°C (Gambuti et al., 2005). Chemical removal relies upon the use of some fining agents, like activated carbon, bentonite, chitin and chitosan, egg albumin, gelatin, oak wood pieces, potassium caseinate, and PVPP (Bellver Soto et al., 2014). Each method shows benefits (cost, simple use, etc...) and drawbacks (effect on color and phenols, etc...), with a removal efficiency ranging from 2 to 98%. Moreover, some agents could cause an adverse reaction in susceptible wine consumers; therefore, the European Commission Directive 2007/68/EC (European Commission [EC], 2007) establishes that all the wines placed on European market, it is compulsory to indicate on the label if they have been treated with adjuncts derived from eggs, fish, and milk.

A promising way for wine decontamination could be the bioremediation (Quintela et al., 2013; Petruzzi et al., 2014e). There two main routes for the bioremediation, i.e., toxin degradation and adsorption. Recently, Abrunhosa et al. (2014) isolated and characterized some strains of *Pediococcus parvulus* able to hydrolize OTA bond by a putative peptidase and produce Ochratoxin α (OT α), a not toxic moiety synthesized by animals and humans as the most important detoxification pathway. A similar mechanism was recovered by De Bellis et al. (2015) for *Acinetobacter calcoaceticus*, isolated from soil. Previously, a OTA-detoxification way was found in *Phenylobacterium immobile* (Wegst and Lingens, 1983), *Trichosporon mycotoxinivorans* (Schatzmayr et al., 2006), *Brevibacterium* spp. (Rodriguez et al., 2011), *Aspergillus* spp. (Bejaoui et al., 2006; Abrunhosa and Venâncio, 2007), and *Rhizophus* spp. (Varga et al., 2005). This pathway is promising; however, the production of OTα could be a threat, because we do not know the implication of the accumulation of this compound in the body.

A second way is OTA adsorption on yeast cell wall throughout fermentation. Toxin could be absorbed by several bacteria (*Lactobacillus rhamnosus*, *L. acidophilus*, *L. plantarum*, *Oenococcus oeni*, *L. brevis*), and fungi (*A. niger*, *A. carbonarius*, *A. japonicus*) as a result of ionic and non-covalent interaction with the cell wall. Petruzzi et al. (2013, 2014a–d, 2015a,b) and Bevilacqua et al. (2014) proposed the yeasts as adsorbing tools both under *in vitro* and *in vivo* conditions. OTA adsorption by yeasts is the result of complex interactions with glucans, and mannoproteins. Moreover, this phenomenon could be strongly affected by some factors, like pH, temperature, sugar, nitrogen supplementation, and to some extent was found partly reversible, as toxin could be released back into wine. An interesting implementation of this approach is yeast entrapment into alginate beads to design a re-usable biocatalyst (Farbo et al., 2016); entrapped yeasts were able to remove the 80% of OTA in 48 h and toxin release by beads could be better controlled than in free cells.

## Fermentation and Post-Fermentation Threats: Biogenic Amines

Biogenic amines are low-molecular-weight organic molecules originated in fermented foods from the microbial catabolism of the corresponding amino acids. Wine BA include histamine (from histidine), tyramine (from tyrosine), tryptamine (from tryptophane), cadaverine (from lysine), and putrescine (from arginine and ornithine). The production of BA is a strategy to obtain metabolic advantages to face certain stress conditions (Wolken et al., 2006). However, this feature seems to be strain-dependent rather than species-specific, suggesting that it could be disseminated by horizontal gene transfer (Lucas et al., 2008; Coton and Coton, 2009).

Although BA are degraded in the human gut lumen by the activity of amino oxidases, adverse health implications can occur in susceptible individuals. Therefore, the intake of high amounts of dietary BA can determine several disorders, from mild symptoms resembling allergic reactions until death in severe cases of histaminosis or tyraminosis (Spano et al., 2010). Moreover, it is crucial to consider the synergistic effect of inhibitors of the amino oxidases such as some drugs, alcohol, or putrescine that act as histamine enhancers. On the other hand, putrescine and other polyamines are involved in cell proliferation, and they have been correlated with cancer events (Spano et al., 2010).

Arginine and histidine are found among the most abundant amino acids in grapes. Therefore, histamine production in wines is a critical concern, since its toxicity could be amplified by the concomitant occurrence of alcohol and high levels of putrescine (Beneduce et al., 2010). Based on this issue, histamine content in wines is differently regulated among European countries, thereby foreclosing certain commercial opportunities for winemakers. Moreover, high levels of putrescine and cadaverine negatively affect the aromatic bouquet of the wine (Beneduce et al., 2010). Finally, less obvious is the threat posed by the intake of live BA-producing bacteria that could contribute to increase the risk of BA formation in the gut environment (Russo et al., 2012).

The production of BA in wine is related to the metabolic activity of lactic acid bacteria (LAB) responsible for the malolactic fermentation (MLF; Lonvaud-Funel, 2001) or associated to spoilage and/or contaminant microorganisms (Benavent-Gil et al., 2016). Since MLF is especially wanted in red wine production, higher BA amounts are usually found in red wines, then in rosè, white, or sparkling wines. *O. oeni* is the main LAB species carrying out the MLF, and some authors reported its capability to produce histamine (Lonvaud-Funel and Joyeux, 1994; Landete et al., 2007; Lucas et al., 2008). However, this metabolic trait of *O. oeni* is controversial and has been recently questioned (García-Moruño and Muñoz, 2012), suggesting that other LAB may be involved in BA formation. Among typical inhabitants of the wine, *Pd. parvulus*, *L. mali*, and *Leuconostoc mesenteroides* are reported to produce histamine, *L. brevis*, *L. hilgardii*, and *L. buchneri* were mainly associated with tyramine and putrescine formation; moreover, some strains could produce two or more BA (Moreno-Arribas et al., 2003; Landete et al., 2007; Coton et al., 2010). Recently, *Enterococcus* spp. isolated from must and wine have been described as tyramine, putrescine, and histamine producers (Capozzi et al., 2011; Pérez-Martín et al., 2014).

Biogenic amines formation during the alcoholic fermentation is considered irrelevant. Accordingly, enological yeasts were found unable to produce BA (Landete et al., 2007). Nonetheless, some strains can produce low amounts of polyamines (Caruso et al., 2002). Moreover, some non-*Saccharomyces* strains were able to synthesize histamine and cadaverine during must fermentation suggesting the importance of a correct yeast management during wine-making (Tristezza et al., 2013).

### Controlling BA in Wine

In fermented foods, different levels of BA can be achieved depending from the occurrence of BA-forming bacteria, the availability of free amino acids and the expression of the corresponding BA biosynthetic pathways under favorable environmental conditions (Spano et al., 2010).

Actually, the control of these toxic compounds in wine is mainly based on the adoption of strategy to prevent their formation than on their elimination from the beverage (Mohedano et al., 2014).

A correct management of all the factors related to the increase of precursor amino acids should be implemented. The abundance of free amino acids is strictly related to agronomical techniques (i.e., nitrogenous fertilization, irrigation, and vintage), environmental conditions, grape variety and/or geographical origin, maturation degree of the grape (Landete et al., 2005; Ancín-Azpilicueta et al., 2008; Del Prete et al., 2009; Cecchini and Morassut, 2010; Ortega-Heras et al., 2014; Smit et al., 2014).

Amino acids are key precursors and contribute to aroma and organoleptic profile of wines. Therefore, it seems to be more advisable to intervene controlling the microflora responsible for the vinification in order to avoid potential BA-producers. Common practices in wine-making are the addition of sulphite and the inoculation of starter cultures in order to inhibit the growth of unknown and uncharacterized indigenous microorganisms. Accordingly, organic wines obtained by spontaneous fermentation and lower levels of sulphite showed higher contents of BA (García-Marino et al., 2010; Comuzzo et al., 2013). In contrast, concurrent yeast/bacteria inoculation of musts has been proposed as an interesting practice to obtain a significant reduction of BA (Izquierdo-Cañas et al., 2012; Smit et al., 2012). Therefore, a remarkable criterion to select oenological starters should be the absence of the genetic determinants to produce BA (Landete et al., 2011; Capozzi et al., 2014). Nonetheless, an underestimated issue is the contamination of commercial yeast starters with BA-producing LAB (Costantini et al., 2009), as well as the interactions between natural yeasts and LAB able to form BA from short peptides (Bonnin-Jusserand et al., 2012).

Wine environment enhance BA formation, since the genes responsible for BA production were induced at low pH (Arena et al., 2011), and BA content could be increased under wine poor nutritional conditions (Aredes-Fernández et al., 2010). Winemaking practices (maceration, aging, and storage) can influence the levels of BA in wine, probably due to an increase of the amino acid concentration from grape skin, yeast autolysis or contact with lees (Marques et al., 2008; Ancín-Azpilicueta et al., 2010; Smit and du Toit, 2013; Smit et al., 2013).

Therefore, prevent BA biosynthesis in wine is not always possible since a number of microbiological, chemical, and physical conditions should be addressed in a way that may affect the organoleptic properties of the wine or result incompatible with specific productions such as spontaneous fermented, organic, or sulphite-free wines. An attractive strategy to correct the occurrence of BA in wines could be the employment of BA-degrading microorganisms (Alvarez and Moreno-Arribas, 2014). Grapevine ecosystem fungi and some wine LAB belonging to *Lactobacillus* and *Pediococcus* genera were able to degrade histamine, tyramine, and putrescine in culture media (García-Ruiz et al., 2011; Cueva et al., 2012). However, the ability of these microorganisms to reduce BA was negatively affected by wine matrix, suggesting that the effectiveness of the amino oxidase activity could be modulated by the physico-chemical wine composition (García-Ruiz et al., 2011; Cueva et al., 2012). With a similar approach, two wine *L. plantarum* were able to reduce tyramine and putrescine in media containing BA or in presence of specific chemical precursors and BA-producers LAB (Capozzi et al., 2012). These strains showed promising technological aptitudes, suggesting that the ability to degrade BA could be a driver to select a new generation of MLF starter cultures (Capozzi et al., 2012). In a recent study, multicopper oxidases from wine-associated LAB have been purified and identified as responsible for the reduction of histamine, tyramine, and putrescine in wine (Callejón et al., 2014). The same authors further investigated this enzyme with a recombinant approach, indicating that oenological LAB or their purified enzymes could solve the problem of high amine concentrations in wine (Callejón et al., 2016). This strategy could be particularly interesting if the employment of a microbial strain is not recommended as starter culture for wine production, as reported for the yeast *Debaryomyces hansenii* although this microorganism was able to degrade a broad spectrum of BA (Bäumlisberger et al., 2015). Accordingly, it was demonstrated that a flavin-dependent oxidase from *Kocuria varians*, a bacterial starter for the manufacture of fermented meat, degraded putrescine and cadaverine even under the harsh wine conditions (Callejón et al., 2015).

## Future Perspectives

Although the biological degradation of BA and the removal of OTA offer interesting perspectives for wine industry, prevention is the most important strategy to control the threat of these toxic compounds. Nowadays, advances in molecular and ohmic approaches may ensure considerable advantages to select safe microbial starter cultures. At the same time, progresses in analytical tools can provide an early detection of BA and OTA encouraging their monitoring during winemaking and storage. However, it is presumable that the risk of these compounds in wine is still underestimated, due to a poor awareness of the consumer, misdiagnosis, and discrepant surveillances across the world countries. In this regard, it is crucial to emphasize the effort of regulatory agencies, such as recently EFSA, to propose a standardized and harmonious framework for BA and OTA risk assessment and detection (European Food Safety Authority [EFSA], 2011).

## Author Contributions

PR, VC, GS, MC, MS, and AB performed an accurate research in the literature and planned paper. PR and AB wrote the paper.

## Conflict of Interest Statement

The authors declare that the research was conducted in the absence of any commercial or financial relationships that could be construed as a potential conflict of interest.
